# Professional Self-Concept and Self-Confidence for Nurses Dealing with COVID-19 Patients

**DOI:** 10.3390/jpm12020134

**Published:** 2022-01-20

**Authors:** Nabeel F. Allobaney, Nidal F. Eshah, Ahmad A. Abujaber, Abdulqadir J. J. Nashwan

**Affiliations:** 1 Department of Nursing, Hazm Mebaireek General Hospital (HMGH), Hamad Medical Corporation (HMC), Doha, Qatar; NAlLobaney@hamad.qa (N.F.A.); aabujaber@hamad.qa (A.A.A.); 2 Faculty of Nursing, Zarqa University, Zarqa, Jordan; nidal2000jo@yahoo.com; 3 Faculty of Nursing, University of Calgary in Qatar (UCQ), Doha, Qatar

**Keywords:** COVID-19, nurses, self-concept, self-confidence, professional

## Abstract

Purpose: To identify the impact of dealing with COVID-19 patients in clinical areas on nurses’ professional self-concept and self-confidence. Background: Professional self-concept is considered a critical factor in the recruitment/retention process in nursing, nursing shortage, career satisfaction, and academic achievements. Professional self-confidence is also a crucial determinant in staff satisfaction, reducing turnover, and increasing work engagement. Design: Descriptive, comparative study. Methods: The study was conducted between February to May 2021 by utilizing a convenience sampling technique. A total of 170 nurses from two facilities were recruited from two COVID-19- and non-COVID-19-designated facilities. The level of professional self-concept and self-confidence was assessed by utilizing the Nurses’ Self-Concept Instrument and Self-Confidence Scale. Results: The professional self-concept level among the group exposed to COVID-19 patients was lower than the comparison group, while the professional self-confidence level among the exposed group to COVID-19 patients was similar to the comparison group. On the other hand, the satisfied staff and those who received professional training in dealing with COVID-19 patients reported a higher level of professional self-concept. Conclusions: Dealing with COVID-19 patients has an impact on professional self-concept; the exposure group was lower than those who did not deal with COVID-19 patients, while the professional self-confidence level among the exposed group was similar to the comparison group. Getting professional training in dealing with COVID-19 patients and being satisfied at work were significant factors in improving professional self-concept. Policymakers should create strategies that target the improvement of professional training in dealing with COVID-19 patients.

## 1. Introduction

Historically, nurses fought against different health crises such as Cholera, H1N1, SARS, and Ebola. By considering healthcare providers’ mental health alteration during diseases outbreak, several organizations released warning notifications about the need for professional and mental health interventions that target healthcare professionals dealing with disease outbreaks and pandemic situations [1].

Currently, the COVID-19 pandemic is causing a significant impact on all nurses worldwide and other healthcare professionals [2,3]. The prevalence of COVID-19 cases has reached more than 304 million proven cases globally and more than five million deaths worldwide [4]. It has been reported that the shortage of personal protective equipment that was available for nurses and increasing the workload increased the chance of transmitting the disease and contracting it [5]. Nurses have significant roles in fighting against the COVID-19 pandemic. They play substantial roles in directing, running, and maintaining healthcare systems and conserving them from collapse [6]. During this pandemic, nurses are facing several clinical challenges such as alterations in working hours, increasing workload, policy changing, alterations in psychological well-being, as well as increased levels of anxiety, stress, depression, and insomnia [1,7,8,9,10,11,12]. Accordingly, nurses dealing with COVID-19 patients are at high risk of facing physical, psychological, and professional consequences.

Self-concept is considered as one of the essential professional determinants. According to Lone and Lone (2016), self-concept is the whole set of attitudes, opinions, and cognitions that a person has of himself. According to Baumeister [13], self-concept is defined as “the individual’s belief about himself or herself, including the person’s attributes and who and what the self is”. The professional self-concept is defined as “an individual’s perception of self as a professional person, which affects different aspects of professional performance” [14]. According to Randle and Arthur (2007), when nurses have a high professional self-concept, they will be more likely to influence patient healthcare positively; conversely, when nurses have a low professional self-concept, they will be more likely to impact patient healthcare negatively. Self-concept is considered a critical determinant of self-behavior, which could also be applied to the professional self-concept and how the nurses could feel themselves from their perspective as nurses [15]. Self-concept is considered one of the crucial elements in the recruitment/retention process in nursing; despite this, few researchers have made efforts to investigate this area of research. However, there is strong evidence that has been found by researchers indicating that self-concept has a significant influence on other important job-related factors such as stress, burnout, and job satisfaction [16].

According to Roy and Andrews 1999, self-concept is defined as the composite of feelings and beliefs about oneself at a specific time and is formed from internal perceptions and perceptions of others’ reactions. According to the Roy Adaptation Model, the human continuously faces environmental stimuli or crises; accordingly, the self-concept is considered as one of the effector subsystems that might lead to an adaptive or ineffective response. In other words, when a person faces an experience or a crisis, he will fight or flight, and that will be affected by many factors, such as self-concept [17].

Self-confidence is another essential professional determinant, which is defined as the belief of a human in his/her capabilities to accomplish a task [18]. According to Martins et al., (2014), self-confidence is considered an important factor in producing a rapid and appropriate response in critical situations. It was documented that a high level of professional self-confidence among nurses enhanced safe nursing practices and patient safety [19,20]. According to Lundberg (2008), a high level of professional self-confidence in nursing is essential for a safe and smooth transition from nursing college to the clinical side and real nursing life [21]. Sak-Dankosky and colleagues (2014), found in their study that self-confidence in higher-educated persons, and those with a higher income, makes them more able to perform innovative work, hence being more successful in their career and actions [22]. This conclusion is supported by another study conducted by Henderson et al. (2016), especially in the relationship between self-confidence and education program. The findings of previous study suggested that the new nurses are not prepared to meet the requirements to handle patients in palliative care [23]. Hence, further appropriate education is required to support the new nurses’ self-confidence growth better. According to Tivener and Gloe (2015), placing nursing students in a crisis situation such as a high-fidelity simulation for cardiopulmonary resuscitation (CPR) and exposing them to such an experience increased the nurses’ student self-confidence [24]. Moreover, nurses with higher professional self-confidence had more positive behaviors toward family members’ presence during a CPR code [25].

### 1.1. Problem Statement

COVID-19 disease is a new pandemic that strikes different world regions. Additionally, very few studies have investigated the impact of dealing with COVID-19 patients on nurses’ professional self-concept and self-confidence. Dealing with COVID-19 patients might contribute to changing nurses’ professional self-concept and professional self-confidence. However, the magnitude, nature, and direction of change are still vague and need exploration.

### 1.2. Significance

Findings of the current study will offer information about the impact of dealing with COVID-19 patients and specifically understand its impact on nurses’ professional self-concept and self-confidence. This could help healthcare leaders to build policies and strategies to support nurses in their humanistic mission. Investigating the consequences of dealing with COVID-19 patients on nurses would help nurse managers and policymakers in estimating the magnitude and direction of change in the level of professional self-concept and professional self-confidence, and this would help in enhancing the recruitment and retention process. Early recognition of change in professional self-concept and professional self-confidence among nurses will offer early indicators to the future plans and consequently empower the staff effectively.

### 1.3. Study Purpose

The purpose of this study is to identify the impact of dealing with COVID-19 patients in clinical areas on nurses’ professional self-concept and professional self-confidence. Specifically, the current study has the following objectives:

- Investigating the level of professional self-concept among nurses dealing with COVID-19 patients.
- Investigating the level of professional self-confidence among nurses dealing with COVID-19 patients.
- Assessing the differences in professional self-concept and professional self-confidence between the nurses who are dealing with COVID-19 patients and those who do not deal with such patients.

### 1.4. Research Questions

- What is the level of professional self-concept and professional self-confidence among nurses who provide direct care to COVID-19 patients?
- Is there a difference in professional self-concept level between nurses who provide direct care to COVID-19 patients and those who do not?
- Is there a difference in professional self-confidence level between nurses who provide direct care to COVID-19 patients and those who do not?
- Are there differences in professional self-concept and professional self-confidence levels among nurses who provide direct care to COVID-19 patients based on their sociodemographics?

### 1.5. Definitions of Variables

#### 1.5.1. Professional Self-Concept

Conceptual definition: “An individual’s attitude about personal professional competence, performance, and worth along a positive-negative dimension” [26]. Operational definition: The professional self-concept was measured by the Nurses’ Self-Concept Instrument (NSCI) [27].

#### 1.5.2. Professional Self-Confidence 

Conceptual definition: “The belief that one can successfully execute a specific activity” [28]. Operational definition: The professional self-confidence was measured by the Self-Confidence Scale-SCS [29].

## 2. Materials and Methods

This study was conducted in two different hospitals in Qatar. First, hospitals leaders’ consent was obtained and informed about the study officially. Then, the instrument was disseminated by utilizing Google forms. Each electronic form came with an opening page, which acted as implied consent.

### 2.1. Design

A descriptive comparative study design was used to answer the research questions during the period from February to May 2021.

### 2.2. Population and Sample

The target population was the registered nurses working in Qatar’s government sector, as they almost have the same salaries, allowances, and working environment. The accessible population was from Hamad Medical Corporation-HMC/Qatar. The sample was distributed into two groups. The first group was the registered nurses who directly dealt with COVID-19 patients, and the second group was the registered nurses who did not deal with COVID-19 patients. A convenience sampling technique was used in order to recruit a total of 170 nurses, whereas the required sample size based on Cohen’s tables was 64 for each group based on a moderate effect size with a power of 80% and Alpha less than 0.05. Moreover, it was increased to 85 for each group to avoid incomplete questionnaires.

### 2.3. Data Collection and Settings

Initially, the researcher prepared a copy of the IRB and the questionnaire. Then, unit leaders were contacted—mainly the head nurses—to inform them and obtain permission to collect the data. Accordingly, the researcher asked the head nurses to provide him with a list of the registered nurses’ E-mail addresses. The researcher visited the staff at the safety huddle “when the day and night shifts get together” to explain the goals of the study, and teach them how to fill out the questionnaire electronically using Google Forms after obtaining permission and E-mail addresses. Each electronic form came with an opening page, which acted as implied consent. Only the main investigator has access to the returned electronic forms, which he is responsible for safeguarding in Microsoft OneDrive—in his HMC account—and on a password-protected USB drive.

The first setting was the inpatient units in Hazem Mebarik General Hospital (HMGH), a member of Hamad Medical Corporation (HMC). HMGH is a COVID-19 facility where the exposure group was located. This hospital includes seven inpatient units, three of them are the original units before the pandemic, and the other four units are created in March 2020 to help fight against the COVID-19 pandemic. These seven units are now assigned for COVID-19 patients with mild-to-moderate clinical situations. Any patient needed oxygen therapy more than a non-rebreather mask was transferred immediately to the intensive care units. In the time being, these units have about 250 registered nurses working in 12 h shifts. Each staff must complete 40 working hours weekly; the remaining hours will be considered overtime.

The second setting was the inpatient units in Rumailah Hospital (RH), a member of HMC; this is a non-COVID-19 facility where the comparison group was located. This hospital included five inpatient units to deal with non-COVID-19 patients. These units have about 170 registered nurses working in 8 h shifts. Each staff member must complete 40 working hours weekly.

### 2.4. Instruments

The demographic data (age, gender, marital status, nationality, education, job position, experience, and the duration of dealing with COVD-19 patients) were collected utilizing a questionnaire which included the Nurses’ Self-Concept Instrument (NSCI) and Self-Confidence Scale (SCS). 

#### 2.4.1. Self-Concept Instrument

The Nurses’ Self-Concept Instrument (NSCI) was developed by Angel, Craven, and Denson, 2012. This instrument was adapted from Cowin Nurses’ Self-Concept Questionnaire (NSCQ) (2002). The NSCI measured nurses’ professional self-concept with a total of 14 items, each item providing eight points on the Likert scale starting from 1 (definitely false) to 8 (definitely true). The scale is divided into four subscales of knowledge, care, leadership, and staff relations. Item scores for each subscale were summed then divided into the number of the subscale’s items, resulting in the average scores comparable across groups. A higher score introduces a stronger endorsement of each subscale item. There are no cut-off points on this scale. With higher scores reflecting a stronger endorsement of each item, the Nurses’ Self-Concept Instrument has been utilized to assess the level of self-concept in many studies; for example, it was implemented in Australia by Angel et al. (2012) to evaluate Australian local and international students’ nurses’ psychometric properties. As reported by Angel et al. (2012), the construct validity was ensured in the adapted instrument by utilizing confirmatory factor analysis. Additionally, the internal consistency reliability was assessed by using Cronbach’s Alpha (ranged between 0.60 to 0.78) [27].

On the other hand, the original tool by Cowin-Nurses’ Self-Concept Questionnaire (NSCQ) (2002) was utilized in different populations such as China and Turkey. The Turkish study was conducted by Zencir G., Zencir M., Khorshid L. (2018) and showed that the Cronbach’s alpha coefficient is located between 0.83 to 0.91 for the six subscales; also, the tool had good construct validity by utilizing the exploratory factor analysis—EFA and confirmatory factor analysis—CFA [30]. The same conclusion was obtained in a Chinese study by Cao X and colleagues in 2012 [31].

#### 2.4.2. Self-Confidence Scale (SCS)

The Self-Confidence Scale-SCS was developed by Hicks (2006); the instrument used to measure nurses’ professional self-confidence in providing care with acute clinical deterioration patients. The SCS measured nurses’ professional self-confidence in four dimensions (1) accurately recognizing deteriorating patients, (2) performing basic physical assessments, (3) identifying basic nursing interventions, and (4) evaluating the effectiveness of the interventions. The items were measured by a five-point Likert scale ranging from 1 (not at all confident) to 5 (very confident). The item scores were summed for a total score. The lower score is 12, and the maximum score is 60. A low score is interpreted as low self-confidence, and a high score indicated high self-confidence, with no cut-off points for this scale. SCS instrument was utilized to assess the level of self-confidence for medical surgical nurses by Hart et al. (2014) [32]. The internal consistency reliability was reported by Hart et al. (2014) using Cronbach’s Alpha (ranging between 0.93 to 0.96). The tool had good construct validity by utilizing factor analysis [29].

### 2.5. Pilot Study

A pilot study was conducted, including 15 participants from both settings, to assess the feasibility of study applicability, clarity of the scales, and the ability to access the settings. Additionally, it was crucial to assess the cooperation of unit leaders, charge nurses, and registered nurses. Additionally, the required time to fill the instrument was estimated, and it was almost 15 min.

### 2.6. Inclusion and Exclusion Criteria

The eligibility criteria included registered nurses—“those who have a minimum of bachelor’s degree in nursing”—and those working in HMC. For the first group, the registered nurses should have been exposed and dealt with COVID-19 patients for at least three months to make sure that the staff had worked with COVID-19 patients directly, not just for a training period or shadowing of any other registered nurses. For the second group, the “control group”, the registered nurses should not have exposed or dealt with COVID-19 patients for the entire period from the start of the COVID-19 pandemic until the collecting of the questionnaire. Moreover, they should have the same working environment as the exposure group.

The exclusion criteria included all registered nurses working with the research participants such as nursing students, those who are registered nurses but handle other positions in the research setting such as quality reviewer or infection control reviewer, and those who are in acting places.

### 2.7. Ethical Approval

The study was conducted in full conformance with principles of the “Declaration of Helsinki”, Good Clinical Practice (GCP), and within the laws and regulations of MoPH in Qatar. Implied consent was obtained from the participants, and the researcher assured voluntary participation for the subjects. Additionally, the questionnaire was disseminated without names or corporation numbers to assure participant anonymity and data confidentiality. Original authors provided the researcher with permission to use the scales. The researcher obtained permission from Zarqa University ethical committee and IRB from HMC (MRC-01-21-076), Qatar, as well.

### 2.8. Statistical Analysis

The Statistical Package for the Social Sciences (SPSS) IBM, version 25 was used to analyze the data. Descriptive statistics, including percentages, frequencies, means, and standard deviations, were used to describe the sample and answer the research questions about the level of self-concept and self-confidence. In addition, Mann–Whitney U and Kruskal–Wallis tests were used to answer the question “Are there significant differences in professional self-concept and professional self-confidence levels among nurses who provide direct care to COVID-19 patients based on their socio-demographics?”. Additionally, the Mann–Whitney U test was used to assess the differences between the groups.

Assumptions were checked and insured before using the inferential statistics. The researcher checked the outliers and the missing values and dealt with them appropriately. Then, the researcher ran the analysis. The data were considered significant when *p*-value < 0.05.

## 3. Results

From 420 invitations sent to HMGH and RH staff nurses (the exposure and comparison group, respectively), 254 responses were sent back to the researcher with a 60.5% response rate. Among those who responded, 170 responses were included in this study, as 18 participants submitted missing data files, and 66 participants from the comparison group were excluded as they dealt with COVID-19 patients for a short period. The remaining is 170 subjects constitute the two groups, the exposure group including 85 participants and the comparison group including 85 participants.

### 3.1. Sample Characteristics

The average age for participants from the two groups was 36 years old (SD ± 6.29) and ranged between 25 and 58 years old. The average clinical experience for participants from both groups was 12.5 years (SD ± 5.75), with a minimum of 2 years and a maximum of 33 years.

Most of the participants in the two groups were females (53.5%), Asian ethnicity (67%), working by permanent contract (95.3%), bachelor degree holders (94,1%), staff nurses (81.2%), working a day–night 12 h shift format (51.2%), satisfied about their role as nurses (69.4%), had not had COVID-19 infection (80%), and (53.5%) did not receive professional training about dealing with COVID-19 patients (Table 1).

### 3.2. Professional Self-Concept and Self-Confidence Scores

The first research question was: What are the scores of professional self-concept and professional self-confidence among the staff nurses? The result showed that the exposure group has an average score of professional self-concept 7.2 out of 8 (SD ± 0.71), compared with 7.5 out of 8 (SD ± 0.43) for the comparison group. The result showed that the average total score of the professional self-confidence for the exposure group was 54.5 (SD ± 6.34), and for the comparison group was 54.6 (SD ± 5.43). The results of NSCI and SCS are presented in Table 2 and Table 3, respectively.

### 3.3. Differences in Professional Self-Concept Based on Exposure to COVID-19 Patients

The second research question was: “Are there significant differences in professional self-concept scores between the nurses who provide direct care to COVID-19 patients and those who do not?” The assumption of normality was violated according to Pearson’s skewness coefficient; therefore, the nonparametric Mann–Whitney-U test was used to compare the professional self-concept scores.

The Mann–Whitney-U test revealed a statistically significant difference between the two groups, the exposure group mean rank = 76.06, and the mean rank for the comparison group = 94.93 (U = 2811, Z = −2.505, *p* < 0.05). Table 4 showed the details regarding the differences in the professional self-concept scores and its subdimensions.

### 3.4. Differences in Professional Self-Confidence Based on Exposure to COVID-19 Patients

The third research question was: Are there significant differences in professional self-confidence level between the nurses who provide direct care to COVID-19 patients and those who do not?

The assumption of normality was violated according to Pearson’s skewness coefficient; therefore, the nonparametric Mann–Whitney-U test was used to compare the professional self-confidence scores. A Mann–Whitney-U test revealed no significant difference between the two groups, the exposure group mean rank = 87.47, and the mean rank for the comparison group = 83.53 (U = 3445, Z = −0.52, *p* = 0.59). Table 5 shows the details regarding the differences in the professional self-confidence scores and its subdimensions.

### 3.5. Differences in Professional Self-Concept and Professional Self-Confidence Based on Nurses’ Sociodemographics

The fourth research question was: Are there significant differences in professional self-concept and professional self-confidence levels among nurses who provide direct care to COVID-19 patients based on their sociodemographics? The assumption of normality was violated according to Pearson’s skewness coefficient; therefore, the nonparametric Kruskal–Wallis test was used to compare the professional self-concept and self-confidence scores.

The researcher reports only professional self-concept scores, as professional self-confidence scores revealed no significant difference between the groups, as illustrated in the previous section.

The Kruskal–Wallis test revealed a statistically significant difference in professional self-concept score based on staff satisfaction about their role as nurses and if they received professional training in dealing with COVID-19 patients as *p* < 0.05 for both dimensions, whereas the Kruskal–Wallis test revealed no statistically significant difference based on age *p* = 0.89, gender *p* = 0.8, marital status *p* = 0.13, ethnicity *p* = 0.56, employment status *p* = 0.69, experience in nursing *p* = 0.49, educational level *p* = 0.07, professional role *p* = 0.36, or duty format *p* = 0.076. For further details about the mean rank differences for professional self-concept, the Mann–Whitney-U test is illustrated in Table 6.

## 4. Discussion

Few studies have investigated the impact of dealing with COVID-19 patients on nurses’ professional self-concept and self-confidence. Dealing with COVID-19 patients might change nurses’ professional self-concept and professional self-confidence. This chapter discusses and highlights the main findings of how professional self-concept and professional self-confidence were affected during the COVID-19 pandemic.

### 4.1. Professional Self-Concept

One of the main interests of this study was to compare groups’ similarities and differences in professional self-concept across exposure and comparison groups. By comparing the results, the exposure group had a lower average of professional self-concept than the comparison groups, especially in the care and knowledge dimensions. The lower average in the exposure group might result from lacking enough time to be prepared ahead of time before the pandemic struck. Additionally, it might be related to working under high pressure, especially those who work at field hospitals, as they do not have enough time to update their knowledge and practices.

This result is consistent with another study conducted by Zang et al. [33]. Their results indicated that the most prevalent problem in their survey was altered self-image with (87.8%) of the screened frontline subjects; these results provide new insight into the relationship between dealing with a crisis such as the COVID-19 pandemic and its impact on frontline healthcare workers, especially nurses.

In contrast with this study, another study indicated that professional self-concept among nursing students in China was high [34]. This might be because the screened subjects were nursing students and had no real experience in the nursing field, and the data were collected during the first stage of this pandemic before facing the consequences. Additionally, it might be related to cultural differences such as race, language, beliefs, economic or educational factors, or values of Chinese nurses.

Moreover, in contrast with this study, another study indicated that the professional identity of intensive care and emergency nurses was greatly improved during the primary stage of the COVID-19 outbreak [9]. This difference in results might be due to differences in the work environment and cultural diversity, as our research setting consists of different nationalities, cultures, believes, and languages [35,36]. On the other hand, Zhang’s study was exclusive to intensive care and emergency nurses, and it was conducted during the first stage of the COVID-19 pandemic.

### 4.2. Professional Self-Confidence

The other main interest of this study was to compare groups’ similarities and differences in professional self-confidence across the exposure and comparison groups. The results indicated that the exposure group had a similar total score of professional self-confidence to the comparison group. This might be because HMC has standard training, competency validation, and revalidation across all HMC facilities, which might decrease the staff practices’ variation and make nurses feel competent.

This study result contrasts with another study conducted by Simonetti et al. (2021). In their study, they found that the prevalence of low self-efficacy among Italian nurses during the COVID-19 pandemic was high [37]. This might be due to the severe epidemiological spread of COVID-19 in Italy, conversely with Qatar.

Moreover, in contrast with this study, in another study conducted by Vagni et al., 2020, they revealed that the frontline staff dealing with the COVID-19 pandemic had low self-efficacy in coping with challenges and threats [38]. This might be due to the severe epidemiological spread of COVID-19 in Italy and might be due to screening many disciplines in the survey such as doctors, nurses, and other healthcare providers.

### 4.3. Deference Based on Sociodemographics

The results indicated that there are no statistically significant differences between the groups based on sociodemographics. This might be because HMC treats all staff from different gender, cultures, religions, and ethnicities professionally and in equity.

This result contrasts with another study conducted by Sabanciogullari and Dogan (2017); their study showed that the professional self-concept was higher with older nurses, females, seniors, and master’s degree holders. This difference may be because the participants were not exposed to a stressful situation such as the COVID-19 pandemic [39].

### 4.4. Deference Based on Work-Related Factors

In addition, this study indicated that the satisfied staff had a higher level of professional self-concept among those who dealt with COVID-19 patients than moderately or dissatisfied staff. This result is consistent with other studies [31,40]. This agreement might be because staff satisfaction is affected by many job-related factors such as stress, burnout, and pressure at work, which are considered expected complications during the COVID-19 pandemic.

Additionally, this study revealed that those who received professional training in dealing with COVID-19 patients had a higher level of professional self-concept than those who did not have such training. This result is consistent with other studies [24,25,37,41,42]. These studies agreed that the trained nurses have higher professional self-confidence than those who did not get such training. This might lead to a high level of professional self-concept as both professional self-confidence and professional self-concept are considered crucial elements of professional identity.

### 4.5. Strengths and Limitations

As far as we know, this is the first study to assess professional self-concept and professional self-confidence among nurses dealing with COVID-19 patients in the state of Qatar. The online survey had excellent access to nurses in the exceptional period of the COVID-19 pandemic. However, it is beyond the scope of this study to address the relationship between professional self-concept and professional self-confidence and how they are affected by staff burnout, clinical behaviors, engaging in health-promoting behaviors, and turnover.

This study’s sample was multicultural and comprised several nationalities. However, ethnicities such as Europeans, Chinese, Russians, and others, were not screened in the survey, as HMC has few staff from these regions.

Additionally, the convenience sampling technique was adopted in our survey. However, considering a probability sampling technique such as simple random sampling will provide more accurate results. Moreover, a longitudinal study design will be required to assess the long-term impact of dealing with COVID-19 patients.

On the other hand, utilizing a more suitable sample and groups close in numbers will allow for establishing parametric statistics and obtaining more powerful results. Additionally, conceptual and theoretical frameworks were not used in this study.

## 5. Conclusions

The professional self-concept level among the group exposed to COVID-19 patients was lower than the comparison group of those who did not deal with COVID-19 patients, while the professional self-confidence level among the group exposed to COVID-19 patients was similar to the comparison group.

On the other hand, the satisfied staff and those who received professional training in dealing with COVID-19 patients reported a higher level of professional self-concept than dissatisfied staff and those who did not receive professional training in dealing with COVID-19 patients.

### Recommendations

It is recommended that healthcare leaders and policymakers improve nurses’ professional self-concept by enhancing staff satisfaction and creating strategies that target professional training in dealing with COVID-19 patients to prepare the staff ahead of time to deal with such pandemics in the future.

In addition, nursing students should be well-prepared for working with highly contagious diseases and dealing with crises such as COVID-19. Therefore, dealing-with-crises courses should be integrated within the nursing curricula of undergraduate students.

On the other hand, future research work is recommended to establish whether staff burnout, clinical behaviors, engaging in health-promoting behaviors, and turnover are factors that affect the professional self-concept or not.

The perceived professional self-concept was low for these frontline nurses, and correlated with satisfaction level and professional training. Therefore, to guarantee nurses’ occupational safety, stable mental health, and staff retention, healthcare leaders should recognize the commitment of frontline staff nurses and pay more attention to staff professional self-concept.

Additionally, healthcare leaders should pay more attention to the staff satisfaction level, screen satisfaction level frequently, address any changes in satisfaction level, and treat the correlated factors.

On the other hand, nursing educators should keep updating staff nurses with new knowledge and protocols to fight against the COVID-19 pandemic. Additionally, nursing educators must explain to frontline staff the expected patient needs that come from the new disease progress and find a way to bring a feeling of enjoyment during patient care. Additionally, nursing educators should train the frontline staff about how to solve nursing problems.

## Figures and Tables

**Table 1:** Participants’ sociodemographic and work-related factors (*n* = 170).

Variables		Exposure Group % (*n*)	Comparison Group % (*n*)	Total Sample % (*n*)
Age	20–29 years old	9.4 (8)	3.5 (3)	6.5 (11)
	30–39 years old	82.4 (70)	60 (51)	71.2 (121)
	40–49 years old	5.9 (5)	24.7 (21)	15.3 (26)
	50–59 years old	2.4 (2)	11.8 (10)	7.1 (12)
Gender	Male	80 (68)	12.9 (11)	46.5 (79)
	Female	20 (17)	87.1 (74)	53.5 (91)
Ethnicity	Asian	77.6 (66)	88.2 (75)	67.1 (114)
	African	2.4 (2)	2.4 (2)	2.4 (4)
	Arabic	17.6 (15)	9.4 (8)	13.5 (23)
	Western	2.4 (2)	0 (0)	1.2 (2)
Contract type	Permanent contract	94.1 (80)	96.5 (82)	95.3 (162)
	Temporary contract	5.9 (5)	3.5 (3)	4.7 (8)
Educational level	Bachelor	92.9 (79)	95.3 (81)	94.1 (160)
	Master	7.1 (6)	4.7 (4)	5.9 (10)
Professional role	Registered Nurse—RN	76.5 (65)	85.9 (73)	81.2 (138)
	Charge Nurse—CN	20 (17)	7.1 (6)	13.5 (23)
	Head Nurse—HN	3.5 (3)	7.1 (6)	5.3 (9)
Experience in nursing	≤10 years	58.8 (50)	30.6 (26)	44.7 (76)
	>10–20 years	38.8 (33)	51.8 (44)	45.3 (77)
	>20 years	2.4 (2)	17.6 (15)	10 (17)
Duty format	Fixed morning	3.5 (3)	10.6 (9)	7.1 (12)
	Day/Night—12 h	87.1 (74)	15.3 (13)	51.2 (87)
	Day/Evening/Night—8 h	9.4 (8)	74.1 (63)	41.8 (71)
Satisfaction level about their role as nurses	Satisfied	62.4 (53)	76.5 (65)	69.4 (118)
	Moderately satisfied	28.2 (24)	22.4 (19)	25.3 (43)
	Dissatisfied	9.4 (8)	1.2 (1)	5.3 (9)
History of getting COVID-19 infection	Yes	27.1 (23)	12.9 (11)	20 (34)
	No	72.9 (62)	87.1 (74)	80 (136)
Receive professional training in dealing with COVID-19	Yes	56.5 (48)	36.5 (31)	46.5 (79)
	No	43.5 (37)	63.5 (54)	53.5 (91)

**Table 2:** Nurses’ professional self-concept and its subdimensions’ scores.

Dimensions		Min	Max	Mean	SD±
Exposure group	Professional self-concept (Average)	3.7	8	7.2	0.71
	Care	5	8	7.2	0.79
	Knowledge	3	8	7.2	0.75
	Staff relations	5.7	8	7.5	0.59
	Leadership	2	8	7	1.18
Comparison group	Professional self-concept (Average)	6.3	8	7.5	0.43
	Care	6.7	8	7.6	0.42
	Knowledge	5.8	8	7.5	0.46
	Staff relations	6.3	8	7.7	0.44
	Leadership	5.3	8	7.3	0.63

**Table 3:** Nurses’ self-confidence and its subdimensions’ scores.

Dimensions		Min	Max	Mean	SD±
Exposure group	Self-Confidence (Total)	31	60	54.5	6.34
	Recognition	6	15	13.5	1.80
	Physical assessments	9	15	14	1.46
	Interventions	7	15	13.6	1.83
	Evaluation	7	15	13.5	1.81
Comparison group	Self-Confidence (Total)	33	60	54.6	5.43
	Recognition	9	15	13.7	1.39
	Physical assessments	9	15	13.9	1.27
	Interventions	8	15	13.6	1.56
	Evaluation	7	15	13.4	1.66

**Table 4:** Differences in professional self-concept and its subdimensions based on exposure to COVID-19 patients.

	Professional Self-Concept (Total)	Care	Knowledge	Staff Relations	Leadership
Mann–Whitney U	2811	2597	2962.5	3203	3222.5
Z	−2.505	−3.26	−2.068	−1.372	−1.236
*p* value	0.01 *	0.001 *	0.03 *	0.17	0.21

* Statistically significant difference.

**Table 5:** Differences in professional self-confidence and its subdimensions based on exposure to COVID-19 patients.

	Confidence Total Score	Recognition Subscale	Physical Assessments Subscale	Interventions Subscale	Evaluation Subscale
Mann–Whitney U	3445	3567	3292	3484.5	3444.5
Z	−0.52	−0.15	−1.07	−0.42	−0.55
*p* value	0.59	0.88	0.28	0.67	0.57

**Table 6:** Pairwise comparisons for the professional self-concept scores.

Variables	Category	*n*	Mean Rank	*p*-Value
Satisfaction about their role as nurses	Dissatisfied	8	15.69	*p* = 0.77
	Moderately satisfied	24	16.77	
	Total	32		
Satisfaction about their role as nurses	Dissatisfied	8	16	*p* > 0.05 *
	Satisfied	53	33.26	
	Total	61		
Satisfaction about their role as nurses	Satisfied	53	44.66	*p* > 0.05 *
	Moderately satisfied	24	26.5	
	Total	77		
Received professional training in dealing with COVID-19	Yes	48	47.92	*p* > 0.05 *
	No	37	36.62	
	Total	85		

* Statistically significant difference.

## Data Availability

All data generated during this study are included in this published article.
